# Plasma proteome correlates of lipid and lipoprotein: biomarkers of metabolic diversity and inflammation in children of rural Nepal[S]

**DOI:** 10.1194/jlr.P088542

**Published:** 2018-11-25

**Authors:** Sun Eun Lee, Kerry Schulze, Christine P. Stewart, Robert N. Cole, Lee S-F. Wu, Abdulkerim Eroglu, James D. Yager, John Groopman, Parul Christian, Keith P. West

**Affiliations:** Center for Human Nutrition* Johns Hopkins Bloomberg School of Public Health, Baltimore, MD; Department of Environmental Health and Engineering,** Johns Hopkins Bloomberg School of Public Health, Baltimore, MD; Program in International and Community Nutrition, † Department of Nutrition, University of California, Davis, CA; Mass Spectrometry and Proteomics Facility, § Johns Hopkins School of Medicine, Baltimore, MD

**Keywords:** plasma proteomics, high density lipoprotein-cholesterol, low density lipoprotein-cholesterol, triglycerides

## Abstract

Proteins involved in lipoprotein metabolism can modulate cardiovascular health. While often measured to assess adult metabolic diseases, little is known about the proteomes of lipoproteins and their relation to metabolic dysregulation and underlying inflammation in undernourished child populations. The objective of this population study was to globally characterize plasma proteins systemically associated with HDL, LDL, and triglycerides in 500 Nepalese children. Abnormal lipid profiles characterized by elevated plasma triglycerides and low HDL-cholesterol (HDL-C) concentrations were common, especially in children with subclinical inflammation. Among 982 proteins analyzed, the relative abundance of 11, 12, and 52 plasma proteins was correlated with LDL-cholesterol (*r* = −0.43∼0.70), triglycerides (*r* = −0.39∼0.53), and HDL-C (*r* = −0.49∼0.79) concentrations, respectively. These proteins included apolipoproteins and numerous unexpected intracellular and extracellular matrix binding proteins, likely originating in hepatic and peripheral tissues. Relative abundance of two-thirds of the HDL proteome varied with inflammation, with acute phase reactants higher by 4∼40%, and proteins involved in HDL biosynthesis, cholesterol efflux, vitamin transport, angiogenesis, and tissue repair lower by 3∼20%. Untargeted plasma proteomics detects comprehensive sets of both known and novel lipoprotein-associated proteins likely reflecting systemic regulation of lipoprotein metabolism and vascular homeostasis. Inflammation-altered distributions of the HDL proteome may be predisposing undernourished populations to early chronic disease.

Lipoproteins, circulating complexes of lipid-bound proteins, play central roles in the transport and metabolism of lipids. They support energy metabolism, cholesterol and phospholipids supply to cell membranes, and fat-soluble vitamin transport. Apolipoproteins are integral constituents that determine the physical properties and the metabolic fate of lipoproteins, stabilizing their structure, shuttling cholesterol and triglycerides throughout the body, acting as ligands for cell surface receptors, and regulating enzymatic activities (1). For example, apoA-I provides major structural support for HDL, activates LCAT, and acts as a ligand for HDL receptors, facilitating HDL-cholesterol (HDL-C) efflux capacity (2). apoB, the major scaffold for LDL and a ligand for LDL receptors, plays an essential role in the delivery of cholesterol to peripheral tissues (3). apoCs and apoE, highly enriched in VLDL and chylomicrons, deliver fatty acids to tissues for energy metabolism and regulate the clearance of triglyceride-rich particles from the plasma (1). Except for apoB, other apolipoproteins are exchangeable between different lipoprotein complexes, while the core structural apolipoproteins confer the unique properties of different classes of lipoproteins (4).

Over the past decade, a more complete, if complex, picture of the roles of lipoproteins has been revealed in proteomics studies, expanding knowledge of the size, diversity, and heterogeneity of protein constituents associated with HDL (4–12), LDL (4, 5, 13, 14), and VLDL (4, 5, 15). The HDL proteome, in particular, includes proteins involved not only in lipid transport and metabolism but also in inflammation, the complement system, protease inhibitors, and wound healing, elucidating broader roles in anti-inflammatory, anti-oxidative, immune-regulatory, and anti-apoptotic capacities (6). Although newly discovered cotransported members of lipoprotein assemblies are less abundant and more transient than apolipoproteins (8), these minor proteins are important for lipoprotein metabolism and function, serving to modulate the atherogenic or cardioprotective properties of lipoproteins (16). Beyond proteins physically carried by lipoproteins, plasma proteomics can identify intracellular or extracellular matrix proteins involved either directly or indirectly in lipoprotein production, secretion, clearance, and a wide range of metabolic actions (17), as lipoproteins constantly interact with cells in hepatic and peripheral tissues, including the immune system.

As proteins are important cargoes of lipoproteins, changes in the composition of proteins can modify the metabolism and function of lipoproteins. For example, inflammation and infections induce multiple alterations in lipid and lipoprotein metabolism (18). Chronic inflammatory diseases and infections are associated with hypertriglyceridemia and low HDL-C, as well as atherosclerosis (19–22), likely affecting the innate immune response, which protects a host from pathogenesis. However, prolonged and unresolved inflammation can impair normal lipoprotein metabolism, partly due to changes in the protein components of lipoproteins, especially in protein-rich HDL particles (18). For example, chronic inflammation may induce marked redistribution of HDL protein subpopulations, including enzymes and lipid transfer proteins, compromising HDL functions (23). Systemic analysis of inflammation-induced changes in proteins metabolically linked to lipoproteins may offer insights into the mechanisms and causes of dysregulated lipid metabolism and dysfunctional lipoproteins that may compromise vascular health.

Compelling epidemiologic and clinical evidence suggests that the metabolic risk factors for cardiovascular disease can be detected and observed to progress throughout childhood (24). However, the family of plasma proteomes of lipoproteins [e.g., direct or indirect correlates of HDL-C and LDL-cholesterol (LDL-C)] in childhood, especially in low-resource societies where subclinical inflammation is common, has rarely been explored. In 2006–2008, we conducted a multipronged health and nutrition assessment of a large population cohort of 6–8-year-old children in the southern rural plains (Terai) of Nepal (25, 26). Typical of the region, the studied children were generally undernourished compared with the World Health Organization reference, but nearly 40% were dyslipidemic, characterized by high triglycerides (≥100 mg/dl) and low HDL-C (<40 mg/dl) (27). In a representative subset of these children, more than 25% showed elevated α-1-acid glycoprotein (AGP), a biomarker of chronic systemic inflammation (28). In the current study of 500 children, we globally characterized plasma proteins correlated with plasma HDL-C, LDL-C, and triglyceride concentrations and evaluated their association with inflammation applying a quantitative proteomics approach.

## MATERIALS AND METHODS

### Study population and field assessments

Children in this proteomics study are a subset of a large child follow-up cohort in the southeastern plains district of Sarlahi, Nepal. Briefly, a community-based cluster-randomized controlled trial of antenatal micronutrient supplementation was conducted between 1999 and 2001 (26). Among more than 4,000 liveborn infants during the trial, we revisited about 3,500 children when they were 6–8 years of age, from 2006 to 2008, to assess their health and nutritional status (27). Field workers collected data on child demographic and anthropometric characteristics, education, dietary and morbidity history in the past week, and household socioeconomic status during home visits. Phlebotomists visited households of children early in the morning and collected venous blood samples in 10 ml sodium heparin vacutainers to prevent blood clots. Collected blood samples were transported on ice to the project’s field laboratory in Sarlahi where plasma samples were separated by centrifugation (1,720 *g* for 15 min). Plasma samples were used for lipid assessments and remaining plasma was aliquoted into cryovials that were stored and shipped in liquid nitrogen tanks to the Center for Human Nutrition laboratory at Johns Hopkins University in Baltimore, MD where they were stored at −80°C until thawed for biochemical assessments and/or proteomics analysis (25). Among 2,130 children who met inclusion criteria of having available plasma samples and complete epidemiological data from the original maternal micronutrient supplementation trial and follow-up study, samples from 1,000 children, balanced across the five original maternal intervention groups (n = 200 in each) were randomly selected for micronutrient and inflammation status assessments (28). Of these, 50% of the samples (n = 100 from each maternal intervention group) were randomly selected for proteomics analysis (29). The original antenatal micronutrient supplementation trial was registered at ClinicalTrials.gov as NCT00115271. Oral informed consent was obtained from the parents of eligible children during the child follow-up due to high illiteracy in the study population. Ethical approval for the original maternal trial and child follow-up study was obtained from the institutional review board at the Johns Hopkins Bloomberg School of Public Health, Baltimore, MD and the Nepal Health Research Council in Kathmandu, Nepal. All methods were carried out in accordance with the principles of the Declaration of Helsinki.

### Lipid and inflammation marker assessments

Assessments of lipid profiles and inflammation markers were previously described in detail (25, 28). Plasma concentrations of total cholesterol, HDL-C, and triglycerides were measured using a Cholestech LDX analyzer (Alere Inc.) by enzymatic colorimetric methods. This instrument has been certified for clinical application by the US Center for Disease Control’s Lipid Standardization Program (Atlanta, GA). The instrument is also robust and thus has been used for point-of-care diagnostic testing of lipid levels in remote areas of rural Nepal. The detectable ranges for total cholesterol, HDL-C, and triglycerides were 100–500, 15–100, and 45–650 mg/dl, respectively. Estimates of plasma LDL-C concentrations were calculated using the Friedewald formula (30). Quality control materials provided by the manufacturer were tested weekly and with the arrival of each new lot of cassette supplies for quality assurance. Plasma AGP was chosen as a biomarker of subclinical inflammation over C-reactive protein (CRP) because AGP is more likely to detect chronic inflammation. For example, in Nepal, AGP identified five times more children with systemic low-grade inflammation than CRP using conventional clinical thresholds (30% of children exhibited AGP >1.0 g/l and 6% of children showed CRP >5 mg/l) (31). AGP was measured using a radial immunodiffusion assay (Kent Laboratories; 0.90 ± 0.1 g/l, CV = 10.0%).

### Proteomics analysis

The processes of immune-depletion of high abundant proteins and MS-based proteomics analysis have been previously described (29). Plasma specimens were depleted of six high abundance proteins (albumin, transferrin, immunoglobulin G, immunoglobulin A, anti-trypsin, and haptoglobin) using a Human-6 Multiple Affinity Removal System LC column (Agilent Technologies). Seven depleted samples randomly chosen from the plasma samples of 500 participants and one masterpool sample, which served as an internal standard (32), were digested at 37°C overnight with trypsin using a 1:10 enzyme to protein ratio. Samples were randomly labeled with eight different isobaric tags for relative and absolute quantification (iTRAQ) reagents, which have reporter ions to be detected for relative quantification, and were incubated at room temperature for 2 h. All labeled samples were combined and 90 μl of the combined peptide sample was dissolved in 4 ml of strong cation exchange loading buffer [25% v/v acetonitrile and 10 mK KH_2_PO_4_ (pH 2.8)]. The sample was fractionated into 24 fractions by strong cation exchange chromatography on an Agilent 1200 capillary HPLC system using a PolySulfoethyl A column. Peptides were loaded on to a reverse-phase nanobore column and eluted using a 2–50% acetonitrile and 0.1% formic acid gradient for 110 min at 300 nl/min. Eluting peptides were sprayed into an LTQ Orbitrap Velos mass spectrometer (Thermo Scientific) and interfaced with a NanoAcquity ultra-HPLC (Waters). Precursors and the fragment ions were analyzed at resolutions of 30,000 and 15,000, respectively. Spectra from full MS scans and fragmented MS/MS scans were extracted with and without deconvolution using Thermo Scientific Xtract software and searched against the RefSeq 40 protein database. Peptides were identified from Mascot (Matrix Science v2.3) searches through the Proteome Discoverer software (v1.3; Thermo Scientific) with a confidence threshold of 5% false discovery rate (FDR). A total of 4,705 nonredundant proteins were detected at least one time among 72 iTRAQ experiments conducted to assess plasma samples of the 500 study children (supplemental Table S1). We included 982 proteins quantified in >10% of all 500 plasma proteins of children (n > 50) in the proteomics analysis.

### Statistical analyses

Standard cut-offs for low or high plasma lipid levels for children were derived from the National Cholesterol Education Program Expert Panel on Cholesterol Levels in Children (33). Estimation of relative abundance of proteins from reporter ion intensities across all obtained spectra have been previously described (32). Linear mixed effects (LME) models were employed to estimate linear associations between each log2 transformed-lipid or lipoprotein concentration as dependent variables and relative abundance of individual plasma proteins as a fixed effect, and each iTRAQ experiment as a random effect. Lipid values below the lower limit of detection were excluded from these analyses. We also adjusted for fasting status of children in all statistical models, as more than one-third of the children in the study were not fasted at the time of blood draw (25). We report summary statistics from the LME models, including number of child plasma samples in which a protein was detected, percent change (%) in lipid or lipoprotein concentration per 2-fold (100%) increase in relative abundance of a protein, *P* value calculated by using a two-sided test of a null hypothesis that there is no association between an individual protein and lipid or lipoprotein, q as FDR-adjusted *P* value to correct multiple comparisons (34), and *r* as a correlation coefficient between measured plasma lipid or lipoprotein concentration and its respective best linear unbiased prediction from the LME models (35). Proteins were considered significantly correlated with an outcome when passing a FDR threshold of 5% (q < 0.05). Because of their large number, we explored correlations between proteins associated with HDL-C concentration by calculating pairwise protein:protein correlation coefficients for proteins quantified in relative abundance within each iTRAQ experiment and averaged values across iTRAQ experiments.

Among proteins correlated with HDL-C, LDL-C, and triglyceride concentrations, we estimated and compared mean differences in relative protein abundance in plasma samples between children with and without inflammation (plasma AGP concentration >1.0 g/l or ≤1.0 g/l), adjusting for potential confounders, including child sex, age, ethnicity, fasting status, and stunting and underweight status (based on height-for-age and weight-for-age z-scores less than −2, respectively, derived from the World Health Organization growth reference for children) (36). *P* values were adjusted to control FDR using the Benjamini-Hochberg method (37). Gene symbols of protein GenInfo identifier (gi) numbers were derived from the Human Genome Organization (HUGO) gene annotation and used in the tables and figures (38). General descriptions of proteins were extracted from the NCBI protein database, UniProt, and in-depth review of literature (39, 40). Data visualization was performed using the Cytoscape (41). The data­sets of lipid profiles and relative abundance of reported proteins in this study are available in supplemental Table S2. All analyses were performed using the R Environment for Statistical Computing (version 3.1.2; R Foundation for Statistical Computing, Vienna, Austria).

## RESULTS

### Lipid profiles and inflammation of children

In this cohort of 500 Nepalese children, median (interquartile range) values of plasma concentrations for total cholesterol, HDL-C, and LDL-C (n = 324) were 111 (100, 127), 27 (21, 33), and 71 (62, 83) mg/dl, respectively, and 89 (66, 118) mg/dl for triglycerides. These medians approximate plasma values less than fifth percentiles for total cholesterol and HDL-C, less than tenth percentile for LDL-C, and greater than ninetieth percentile for triglycerides of distributions among child populations between the ages of 6 and 9 in the United States (42, 43). The prevalence of high total cholesterol (≥200 mg/dl) and high LDL-C (≥130 mg/dl) was 0.4% and 0.6%, while the prevalence of high triglycerides (≥100 mg/dl) and low HDL-C (<40 mg/dl) was 40% and 90%, respectively. About 30% of children had inflammation indicated by elevated plasma AGP (>1.0 g/l). Child characteristics including lipid and lipoprotein profiles are compared by inflammation status in **Table 1**. Children with inflammation had lower plasma HDL-C (25 vs. 28 mg/dl; *P* = 0.005) and higher plasma triglyceride (97 vs. 87 mg/dl; *P* = 0.032) concentrations than children without inflammation, while there were no significant differences in plasma concentrations of total cholesterol and LDL-C. Stunting (48% vs. 36%; *P* = 0.015), underweight (57% vs. 45%; *P* = 0.017), and reported episodes of fever (16.8% vs. 4.6%; *P* < 0.001) and diarrhea (7.4% vs. 1.4%; *P* = 0.001) in the past week were more common in children with inflammation than without inflammation. No differences were observed in child demographic and educational characteristics, economics of household, and dietary history between the two groups.

**TABLE 1.** Characteristics of 6- to 8-year-old children in rural Nepal by inflammation status (n = 500)

Child Characteristics	Inflammation*^a^* (n = 149)	No Inflammation*^a^* (n = 351)	*^b^*
Demographic			
Male, %	52.3	48.7	0.519
Age, years	7.5 (0.4)	7.4 (0.4)	0.109
Ethnicity			
Pahadi (vs. Madheshi),*^c^* %	29.5	32.8	0.545
Anthropometry*^d^*			
Height, cm	113.6 (6.0)	114.1 (5.4)	0.372
Weight, kg	18.0 (2.3)	18.4 (2.2)	0.209
BMI, kg/m^2^	13.9 (1.0)	14.0 (1.0)	0.315
Stunting,*^e^* %	47.7	35.5	0.015
Underweight,*^e^* %	57.0	44.9	0.017
Low BMI,*^e^* %	14.8	16.9	0.646
Education, %			
Ever sent to school	67.8	66.4	0.841
Literacy	14.8	18.5	0.377
Economics of household, %			
Electricity	51.0	51.0	1.000
Land ownership	77.2	76.9	1.000
Diet in the past 7 days (≥3 times),*^f^* %			
Bhat (rice)	98.9	100.0	0.451
Corn and wheat	61.8	67.1	0.307
Milk and curd	60.4	53.3	0.173
Fish, chicken, and other meat	14.8	20.2	0.190
Fruit*^g^*	40.3	39.9	1.000
Dark green leafy vegetables	34.9	31.3	0.500
Oil and ghyu (butter)	100	99.7	1.000
Morbidity in the past 7 days (≥1 time), %			
Fever	16.8	4.6	<0.001
Diarrhea	7.4	1.4	0.001
Productive cough	4.0	3.7	1.000
Rapid breathing and grunting	3.4	2.6	0.846
Lipid or lipoprotein concentrations*^h^*			
Total cholesterol, mg/dl	107 (100, 124)	112 (100, 128)	0.165
HDL-C, mg/dl	25 (20, 31)	28 (22, 34)	0.005
LDL-C, mg/dl	71 (64, 81)	72 (62, 83)	0.835
Triglycerides, mg/dl	97 (69, 120)	87 (65, 114)	0.032

Inflammation was defined as plasma AGP concentration >1.0 g/l.

a Values are mean (SD), percentages, or median (interquartile range).

b *P* values were calculated using *t*-test for continuous variables with normal distributions, Mann-Whitney test for continuous variables with skewed distributions, and chi-square test for categorical variables.

c Pahadi pertains to ethnic groups that originated in the Hills of Nepal; Madheshi pertains to groups whose origin is the southern plains (Tarai) of Nepal.

d One outlier in weight (>45 kg) and two outliers in height (>140 cm) and BMI (>20 or <10 kg/m^2^) were excluded.

e The z-scores were calculated based on the World Health Organization reference for 5–19 years (36). Stunting, height-for-age z-score less than −2; underweight, weight-for-age z-score less than −2; and low BMI, BMI-for-age z-score less than −2.

f Data are missing for rice (n = 1), milk and curd (n = 2), and fruit (n = 23).

g Fruit includes mango, papaya, jackfruit, guava, and orange.

h The lowest detectable values were used for values of HDL-C (n = 36), triglycerides (n = 28), and total cholesterol (n = 146) below detection limits. Data are missing for LDL-C (n = 176).

### Plasma proteins correlated with LDL-C concentrations

Eleven proteins were positively correlated with LDL-C (**Table 2**). apoB was most strongly correlated with plasma LDL-C (*r* = 0.69; q = 8.84 × 10^−47^). LDL-C concentrations increased by 121% per 100% increase in relative abundance of apoB. The rest of the proteins were mostly positive associates (*r* = 0.18∼0.58; q = 3.74 × 10^−2^ to 4.41 × 10^−7^), including kinesin family member 20B, platelet activating factor acetylhydrolase (also known as LDL-C-associated phospholipase 2), cholesteryl ester transfer protein (CETP), proteoglycan 4, and apoM, and four intracellular proteins observed in <100 children. Chondroadherin, which regulates chondrocyte growth, was the only protein negatively associated with LDL-C (*r* = −0.42; q = 2.77 × 10^−2^).

**TABLE 2.** Proteins correlated with plasma LDL-C concentrations in children of rural Nepal aged 6–8 years (n = 324)

Protein Name	n*^a^*	*r*	*R*^2^	*P*	q	Percent Change*^b^*	Accession*^c^*	Reference*^d^*
apoB	324	0.69	0.48	6.39 × 10^−50^	8.84 × 10^−47^	120.8	105990532	(5, 13, 14, 53, 76–78)
Kinesin family member 20B (KIF20B)	208	0.47	0.22	6.38 × 10^−10^	4.41 × 10^−7^	37.2	46049114	
Platelet-activating factor acetylhydrolase (PAF-AH)	266	0.30	0.09	4.49 × 10^−7^	1.55 × 10^−4^	25.7	189095271	(5, 13)
G2/mitotic-specific cyclin B3 (CCNB3)	83	0.58	0.34	1.59 × 10^−6^	3.14 × 10^−4^	34.5	90669307	
Zinc finger and BTB domain containing 1 (ZBTB1)	74	0.58	0.33	4.29 × 10^−6^	6.70 × 10^−4^	50.8	182509178	
Netrin receptor UNC5C (UNC5C)	91	0.46	0.21	4.36 × 10^−6^	6.70 × 10^−4^	38.3	16933525	
CETP, plasma	319	0.21	0.05	1.24 × 10^−4^	1.01 × 10^−2^	19.7	169636439	
Proteoglycan 4 (PRG4)	260	0.23	0.05	2.50 × 10^−4^	1.79 × 10^−2^	27.0	189181724	
Chondroadherin (CHAD)	86	−0.42	0.18	4.61 × 10^−4^	2.77 × 10^−2^	−23.4	153251229	
Poly (ADP-ribose) glycohydrolase (PARG)	92	0.36	0.13	5.50 × 10^−4^	2.99 × 10^−2^	25.1	70610136	
apoM	324	0.18	0.03	8.38 × 10^−4^	3.74 × 10^−2^	24.7	22091452	(5, 14, 53, 76, 77)

Eleven proteins quantified by MS and estimated by LME modeling in >10% of the samples that were positively and negatively correlated with LDL-C adjusting for fasting status (q < 0.05), listed by the strength of association (in increasing order of q).

a Number of child plasma samples in which each protein was detected and quantified by MS. Children with no LDL-C data due to under the detectable range of HDL-C (<15 mg/dl), total cholesterol (<100 mg/dl), or triglyceride (<45 mg/dl) were excluded (n = 176).

b Percent change in LDL-C concentrations of children per 100% (two times) increase in relative abundance of a protein.

c GenInfo sequence number as assigned to all protein sequences by the NCBI at the National Library of Medicine, National Institutes of Health.

d Proteins have been previously reported to be physically associated with LDL particles (79).

### Plasma proteins correlated with triglyceride concentrations

Three apoC proteins were most highly correlated with triglycerides (apoC-II/C-III/C-IV) (*r* = 0.51∼0.54; q = 8.24 × 10^−13^ to 7.22 × 10^−17^) (**Table 3**). Other positive correlates included cathelicidin antimicrobial peptide, proteoglycan 4, retinol-binding protein 4 (RBP4), and apoE. Five negatively associated proteins included extracellular matrix-related proteins, such as anthrax toxin receptor (ANTXR)2, neuropilin 1, and insulin-like growth factor binding protein 1, and lipid transfer proteins such as CETP and phospholipid transfer protein (PLTP).

**TABLE 3.** Proteins correlated with plasma triglyceride concentrations in children of rural Nepal aged 6–8 years (n = 472)

Protein Name	n*^a^*	*r*	*R*^2^	*P*	q	Percent Change*^b^*	Accession*^c^*	Reference*^d^*
apoC-II	472	0.54	0.29	4.20 × 10^−20^	7.22 × 10^−17^	47.2	32130518	(5, 53)
apoC-III	472	0.53	0.29	3.31 × 10^−19^	2.84 × 10^−16^	48.8	4557323	(5, 15, 53)
apoC-IV	472	0.51	0.26	1.44 × 10^−15^	8.24 × 10^−13^	37.8	4502161	(5, 15, 53)
Cathelicidin antimicrobial peptide (CAMP)	394	0.44	0.19	1.18 × 10^−6^	5.07 × 10^−4^	25.1	39753970	(53)
Proteoglycan 4 (PRG4)	380	0.40	0.16	7.07 × 10^−6^	2.43 × 10^−3^	49.3	189181724	
ANTXR2	346	−0.40	0.16	1.85 × 10^−5^	5.28 × 10^−3^	−28.3	50513243	(53)
CETP, plasma	465	−0.40	0.16	2.33 × 10^−5^	5.71 × 10^−3^	−23.9	169636439	(53)
Neuropilin 1 (NRP1)	432	−0.39	0.15	5.14 × 10^−5^	1.10 × 10^−2^	−25.4	66864913	
RBP4	472	0.40	0.16	2.45 × 10^−4^	3.91 × 10^−2^	30.1	55743122	
apoE	472	0.40	0.16	2.45 × 10^−4^	3.91 × 10^−2^	30.9	4557325	(53)
PLTP	445	−0.39	0.15	2.73 × 10^−4^	3.91 × 10^−2^	−27.9	5453914	(5, 15, 53)
Insulin-like growth factor binding protein 1 (IGFBP1)	417	−0.33	0.11	3.16 × 10^−4^	4.17 × 10^−2^	−13.2	4504615	

Twelve proteins quantified by MS and estimated by LME modeling in >10% of the samples that were positively and negatively associated with plasma triglycerides adjusting for fasting status (q < 0.05), listed by the strength of association (in increasing order of q).

a Number of child plasma samples in which each protein was detected and quantified by MS. Children with triglyceride concentrations below a detection limit (<45 mg/dl) were excluded (n = 28).

b Percent change in plasma triglycerides of children per 100% (two times) increase in relative abundance of a protein.

c GenInfo sequence number as assigned to all protein sequences by the NCBI at the National Library of Medicine, National Institutes of Health.

d Proteins have been previously reported to be physically associated with VLDL particles.

### Plasma proteins correlated with HDL-C concentrations

**Fig. 1.**
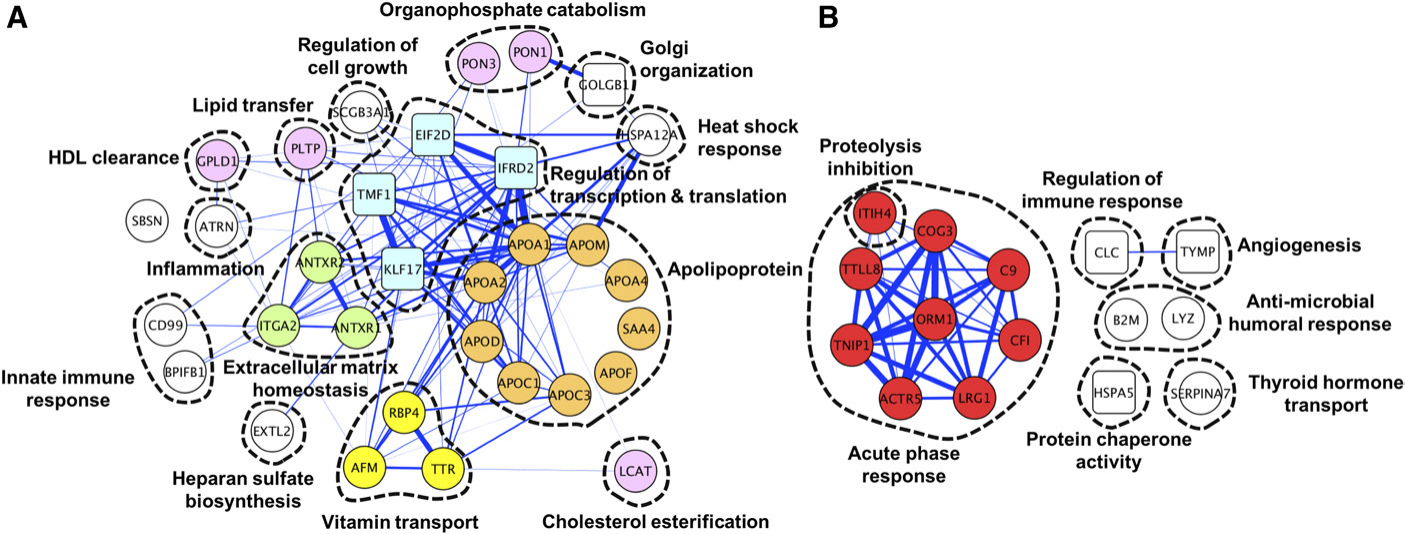
Correlations among plasma proteins positively correlated (A) and negatively correlated (B) with plasma HDL-C concentrations (q < 0.05 and n > 100) in children of rural Nepal aged 6–8 years. The thickness of the lines reflects the strength of positive correlation coefficients between proteins (*r* ≥ 0.3 are presented). Circles and squares represent individual extracellular and intracellular proteins, respectively, indicated as their HUGO gene symbols. Dashed lines indicate molecular or functional clusters. Proteins with colors are mentioned in the text.

Thirty-six proteins were positively correlated with HDL-C (**Table 4**). apoA-I (*r* = 0.79; q = 1.06 × 10^−91^) and apoA-II (*r* = 0.58; q = 4.16 × 10^−21^) were two of the three most strongly associated proteins followed by seven other apolipoproteins [apoA-IV/apoC-I/apoC-III/apoD/apoM/apoF/serum amyloid A4 (SAA4)] (*r* = 0.45∼0.56; q = 4.23 × 10^−3^ to 2.72 × 10^−16^). Proteins with well-defined roles in HDL metabolism and function included PLTP and enzymes, such as LCAT and paraoxonase (PON)1 and PON3. Interestingly, intracellular proteins, such as interferon-related developmental regulator 2 (IFRD2), eukaryotic translation initiation factor 2D (EIF2D), Kruppel-like factor 17 (KLF17), and TATA element modulatory factor 1 (TMF1), were also strongly correlated with HDL-C. Proteins mediating cell-cell and cell-extracellular matrix interaction, such ANTXR1 and ANTXR2, were also among positive correlates. Associated fat-soluble vitamin transporters included retinol binding protein 4, transthyretin, and afamin. Sixteen proteins (**Table 5**), moderately negatively correlated with HDL-C (*r* = −0.33 ∼−0.49; q = 2.05 × 10^−2^ to 7.59 × 10^−4^), are primarily involved in the acute phase response (i.e., orosomucoid 1 or AGP and leucine-rich α-2-glycoprotein 1) and the complement system (i.e., complement component 9 and complement factor I). Protein-protein correlations as well as molecular network and functional clusters of the plasma HDL-C proteome are visualized in **Fig. 1**A and B. Prominent correlations among proteins positively associated with HDL-C (Fig. 1A) were found between apoA-I and apoA-II (*r* = 0.75, colored in orange) and apoA-I with other intracellular proteins, such as IFRD2, EIF2D, KLF17, and TMF1 (*r* = 0.53∼0.91, colored in blue). Among negative HDL-C correlates (Fig. 1B), proteins involved in the acute phase response proteolytic inhibition were strongly correlated with each other (*r* = 0.36∼0.90, colored in red). Correlates of HDL-C appear to be involved in a wide range of functions that extend beyond known functions of HDL, such as regulation of gene transcription and translation and extracellular matrix homeostasis.

**TABLE 4.** Proteins positively correlated with plasma HDL-C concentrations in children of rural Nepal aged 6–8 years (n = 464)

Protein Name	n*^a^*	*r*	*R*^2^	*P*	q	Percent Change*^b^*	Accession*^c^*	Reference*^d^*
apoA-I	464	0.79	0.62	7.68 × 10^−95^	1.06 × 10^−91^	183.8	4557321	(7, 10–12, 60, 80–91)
IFRD2	450	0.64	0.41	3.59 × 10^−33^	2.47 × 10^−30^	63.9	197333755	
apoA-II	464	0.58	0.34	9.04 × 10^−24^	4.16 × 10^−21^	76.9	4502149	(7, 10–12, 60, 80–85, 87–91)
apoD	464	0.56	0.31	8.22 × 10^−19^	2.72 × 10^−16^	53.2	4502163	(7, 12, 60, 80–85, 87–91)
EIF2D	238	0.57	0.33	9.85 × 10^−19^	2.72 × 10^−16^	61.0	56699485	
apoC-I	464	0.51	0.26	1.44 × 10^−13^	2.83 × 10^−11^	18.6	4502157	(7, 10, 11, 60, 80–85, 87–91)
apoM	464	0.51	0.26	7.09 × 10^−13^	1.22 × 10^−10^	54.8	22091452	(7, 10–12, 60, 80–82, 84, 85, 87–91)
PLTP	438	0.51	0.26	1.40 × 10^−10^	2.15 × 10^−8^	50.3	5453914	(7, 60, 80, 81, 87, 88, 91)
ANTXR2	337	0.51	0.26	1.71 × 10^−10^	2.36 × 10^−8^	42.8	50513243	(60)
TMF1	228	0.54	0.29	4.74 × 10^−9^	5.95 × 10^−7^	33.0	110347443	
KLF17	258	0.50	0.25	6.99 × 10^−9^	8.04 × 10^−7^	26.4	104294874	
PON1	464	0.49	0.24	8.81 × 10^−9^	9.35 × 10^−7^	27.3	19923106	(7, 11, 12, 60, 80–85, 87–91)
ANTXR1	315	0.45	0.21	2.39 × 10^−7^	2.36 × 10^−5^	30.0	14149904	(60)
SAA4	459	0.47	0.22	7.79 × 10^−7^	7.17 × 10^−5^	23.9	10835095	(7, 10, 60, 80, 81, 83–85, 87–91)
PON3	457	0.46	0.21	1.23 × 10^−6^	1.06 × 10^−4^	28.1	29788996	(7, 60, 80, 81, 88, 91)
BPI fold containing family B member 1 (BPIFB1)	189	0.52	0.27	1.91 × 10^−6^	1.55 × 10^−4^	26.6	40807482	(60)
apoF	464	0.47	0.22	3.96 × 10^−6^	3.03 × 10^−4^	24.9	4502165	(7, 60, 80–83, 85, 88–91)
Glycosylphosphatidylinositol specific phospholipase D1 (GPLD1)	464	0.46	0.21	9.82 × 10^−6^	6.73 × 10^−4^	31.6	29171717	(60, 80, 82)
Secretoglobin, family 3A member 1 (SCGB3A1)	279	0.54	0.29	1.02 × 10^−5^	6.73 × 10^−4^	14.7	50363226	(91)
LCAT	464	0.45	0.20	2.94 × 10^−5^	1.69 × 10^−3^	52.3	4557892	(7, 60, 80, 87, 88, 91)
apoC-III	464	0.45	0.20	3.16 × 10^−5^	1.74 × 10^−3^	15.5	4557323	(7, 10–12, 60, 80–91)
RBP4	464	0.45	0.21	4.47 × 10^−5^	2.37 × 10^−3^	23.4	55743122	(7, 80, 82, 84, 85, 87, 88, 90)
CD99 molecule (CD99)	444	0.41	0.17	4.78 × 10^−5^	2.39 × 10^−3^	17.1	171543879	
DNA repair and recombination protein RAD54B (RAD54B)	98	0.54	0.29	7.95 × 10^−5^	3.66 × 10^−3^	41.2	6912622	
apoA-IV	464	0.45	0.20	9.49 × 10^−5^	4.23 × 10^−3^	18.4	71773110	(7, 10–12, 60, 80–91)
Transthyretin (TTR)	464	0.45	0.20	1.02 × 10^−4^	4.42 × 10^−3^	32.3	4507725	(7, 10–12, 80–82, 84–86, 88, 90, 91)
Integrin α2 (ITGA2)	175	0.50	0.25	1.32 × 10^−4^	5.51 × 10^−3^	31.1	116295258	(60)
Exostosin-like glycosyltransferase 2 (EXTL2)	301	0.45	0.21	2.10 × 10^−4^	7.82 × 10^−3^	21.0	14149609	
Golgin B1 (GOLGB1)	322	0.44	0.20	2.28 × 10^−4^	8.28 × 10^−3^	19.5	148596984	
Attractin (ATRN)	464	0.45	0.20	2.94 × 10^−4^	1.04 × 10^−2^	34.7	21450863	
Heat shock 70 kDa protein 12A (HSPA12A)	107	0.47	0.22	6.29 × 10^−4^	2.01 × 10^−2^	43.2	119874213	
Afamin (AFM)	464	0.44	0.19	7.23 × 10^−4^	2.17 × 10^−2^	21.2	4501987	(80, 82, 87, 90)
Sushi, von Willebrand factor type A, EGF and pentraxin domain-containing protein 1 (SVEP1)	69	0.61	0.37	7.86 × 10^−4^	2.31 × 10^−2^	46.4	148886654	
Aldehyde dehydrogenase 9 family member A1 (ALDH9A1)	85	0.53	0.28	1.11 × 10^−3^	2.99 × 10^−2^	19.9	115387104	
Suprabasin (SBSN)	121	0.53	0.28	1.88 × 10^−3^	4.42 × 10^−2^	39.5	38348366	
Plexin domain-containing protein 1 (PLXDC1)	94	0.59	0.35	2.26 × 10^−3^	4.91 × 10^−2^	29.4	5011862	

Thirty-six proteins quantified by MS and estimated by LME modeling in >10% of the samples that were positively associated with HDL-C adjusting for fasting status (q < 0.05), listed by the strength of association (in increasing order of q).

a Number of child plasma samples in which each protein was detected and quantified by MS. Children with plasma HDL-C concentrations below a detection limit (<15 mg/dl) were excluded (n = 36).

b Percent change in plasma HDL-C of children per 100% (two times) increase in relative abundance of a protein.

c GenInfo sequence number as assigned to all protein sequences by the NCBI at the National Library of Medicine, National Institutes of Health.

d Proteins have been previously reported to be physically associated with HDL particles (79).

**TABLE 5.** Proteins negatively correlated with plasma HDL-C concentrations in children of rural Nepal aged 6–8 years (n = 464)

Protein Name	n*^a^*	*r*	*R*^2^	*P*	q	Percent Change*^b^*	Accession*^c^*	Reference*^d^*
Complement component 9 (C9)	464	−0.45	0.20	1.28 × 10^−5^	7.59 × 10^−4^	−20.7	4502511	(7, 60, 82, 87, 90)
Orosomucoid 1 (ORM1)	464	−0.45	0.20	6.89 × 10^−6^	4.50 × 10^−4^	−15.7	167857790	(80, 85, 87–91)
Galectin 10 (CLC)	377	−0.48	0.23	3.85 × 10^−5^	2.09 × 10^−3^	−7.0	20357559	
Complement factor I (CFI)	464	−0.44	0.20	1.92 × 10^−4^	6.96 × 10^−3^	−25.3	119392081	(82, 87)
Leucine-rich α-2-glycoprotein 1 (LRG1)	464	−0.44	0.19	8.98 × 10^−5^	4.04 × 10^−3^	−15.5	16418467	(80, 87)
Beta-2-microglobulin (B2M)	457	−0.43	0.18	7.81 × 10^−4^	2.22 × 10^−2^	−13.1	4757826	(80, 87, 88)
Fatty acid binding protein 12 (FABP12)	77	−0.37	0.14	1.18 × 10^−3^	3.14 × 10^−2^	−9.2	157427691	
Thymidine phosphorylase (TYMP)	315	−0.44	0.19	1.81 × 10^−3^	4.03 × 10^−2^	−13.0	4503445	
Tubulin tyrosine ligase-like family member 8 (TTLL8)	272	−0.33	0.11	2.15 × 10^−4^	7.40 × 10^−3^	−19.3	122937293	
TNFAIP3 interacting protein 1 (TNIP1)	356	−0.45	0.20	8.82 × 10^−4^	2.45 × 10^−2^	−13.6	116256481	
Lysozyme (LYZ)	457	−0.44	0.19	1.07 × 10^−3^	2.92 × 10^−2^	−15.0	4557894	(84, 87)
Inter-α-trypsin inhibitor heavy chain family member 4 (ITIH4)	464	−0.45	0.20	1.25 × 10^−3^	3.19 × 10^−2^	−27.2	31542984	(7, 11, 60, 80, 82, 86, 87)
Conserved oligomeric Golgi complex subunit 3 (COG3)	202	−0.49	0.24	1.69 × 10^−3^	4.02 × 10^−2^	−12.4	13899251	
Heat shock 70 kDa protein 5 (glucose-regulated protein, 78 kDa) (HSPA5)	458	−0.42	0.17	2.01 × 10^−3^	4.30 × 10^−2^	−19.1	16507237	
Thyroxine-binding globulin (SERPINA7)	464	−0.44	0.19	1.70 × 10^−3^	4.02 × 10^−2^	−19.5	205277441	(87)
Actin-related protein 5 (ACTR5)	233	−0.42	0.17	7.08 × 10^−4^	2.05 × 10^−2^	−13.7	151301041	

^a^Sixteen proteins quantified by MS and estimated by LME modeling in >10% of the samples that were negatively associated with HDL-C adjusting for fasting status (q < 0.05), listed by the strength of association (in increasing order of q).

a Number of child plasma samples in which each protein was detected and quantified by MS. Children with plasma HDL-C concentrations below a detection limit (<15 mg/dl) were excluded (n = 36).

b Percent change in plasma HDL-C of children per 100% (two times) increase in relative abundance of a protein.

c GenInfo sequence number as assigned to all protein sequences by the NCBI at the National Library of Medicine, National Institutes of Health.

d Proteins have been previously reported to be physically associated with HDL particles (79).

### Changes in relative abundance of proteins correlated with LDL-C, triglyceride, and HDL-C concentrations by inflammation status

**Fig. 2.**
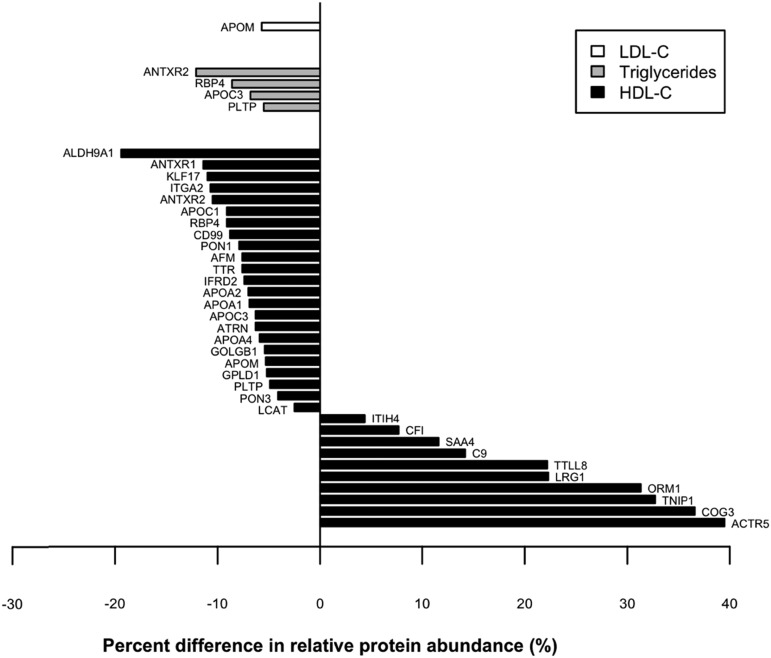
Percent difference in relative abundance of plasma proteins correlated with plasma LDL-C (white), triglyceride (gray), and HDL-C (black) concentrations in children with inflammation (plasma AGP concentration >1.0 g/l) compared with children without inflammation (AGP ≤1.0 g/l), adjusted for child sex, age, ethnicity, stunting, and underweight and fasting status (Benjamini-Hochberg corrected *P* value <0.05).

The relative abundance of proteins associated with the investigated lipids and lipoproteins was compared in children with inflammation (plasma AGP >1.0 g/l) to children without inflammation (AGP ≤1.0 g/l) (**Fig. 2**). Among proteins correlated with HDL-C, 10 were 4∼40% more abundant and 23 proteins were 3∼20% less abundant in children with inflammation, after adjusting for child sex, age, ethnicity, stunting, and underweight status and fasting status. Most proteins with higher and lower relative abundance in the presence of inflammation are negative and positive correlates of HDL-C, respectively. Thus, major structural proteins of HDL (apoA-I and apoA-II) and proteins involved in HDL biosynthesis (i.e., LCAT), vitamin transport (i.e., RBP4), protection against oxidative stress (i.e., PON1), and angiogenesis and tissue repair (i.e., ANTXR1 and ANTXR2), among others, were less abundant in children with inflammation than those without inflammation. An exception was SAA4, an apolipoprotein that was positively correlated with HDL-C (Table 4), but also a positive acute phase reactant, that was 12% higher in inflamed children. Among LDL and triglyceride proteomes, relative abundance of one (apoM) and four proteins (i.e., ANTXR2, RBP4, apoC-III, and PLTP), respectively, were reduced in children with inflammation compared with children without inflammation.

## DISCUSSION

In this southern plains district of Nepal, reflecting undernourished living conditions for about 60% of the country’s population and that typify a wider rural region of Gangetic South Asia, abnormal lipid and lipoprotein profiles were common in young school-aged children, characterized by elevated plasma triglycerides, low HDL-C concentrations, and subclinical inflammation. Our untargeted plasma proteomics approach revealed distinct distributions of 11, 12, and 52 plasma proteins systemically associated with LDL-C, triglyceride, and HDL-C concentrations, respectively, whose functions extend beyond canonical lipid transport and metabolism. Inflammation was associated with remarkable differences in relative protein abundance, especially among biomarkers linked to HDL-C. These results collectively suggest that the family of plasma proteomes correlated with circulating lipoprotein concentrations in children comprise heterogeneous sets of proteins of known and novel biological function, and which are likely affected along a gradient of subclinical inflammation. The results of this large population study also suggest that many proteins not necessarily carried by lipoproteins reflect systemic regulation of lipoprotein metabolism.

The proteome of LDL-C in Nepalese children reveals a unique protein composition dominated by apoB. The strong positive correlation of apoB with LDL-C is consistent with evidence that apoB accounts for about 95% of the total protein mass in a LDL particle (44). Among the remaining proteins, kinesin family member 20B was the second strongest correlate of LDL-C, a motor protein known to transport intracellular vesicles along microtubules (45). A previous study showed elevated levels of serum cholesterol in kinesin family member 13B knockout mice, elucidating its novel role in the regulation of blood cholesterol by facilitating endocytosis of cell membrane receptor-binding LDL in hepatocytes (46). To our knowledge, this is the first human study to show that abundance of the same kinesin family member in plasma is positively correlated with plasma LDL-C concentrations, supporting a putative role of this cytoskeletal protein in regulating LDL clearance. Platelet-activating factor acetylhydrolase, which was previously reported to be physically carried by LDL particles (Table 2) (5, 13), was the third strongest correlate of LDL-C in this study. It modulates pro-inflammation and oxidative stress reactions (47), and has been identified as a risk factor for coronary heart disease (48, 49). Another positive correlate, CETP, participates in LDL remodeling from VLDL by transferring cholesteryl esters from HDL to VLDL particles in an exchange for triglyceride to HDL (50). LDL-C was also correlated with intracellular proteins involved in axon guidance (netrin receptor UNC5C), control of cell cycle (cyclin B3), and DNA damage response (zinc finger and BTB domain-containing protein 1), although their tissue origins and physical and functional relationships with LDL particles are unclear.

Proteins strongly correlated with triglycerides may represent proteins directly associated with triglyceride-rich lipoproteins. For example, apoC-II/C-III/C-IV and apoE are known to reside within VLDL and chylomicrons and play important roles in the metabolism of the triglyceride-rich lipoproteins by regulating activity of lipoprotein lipase and their uptake by peripheral tissues (1). Cathelicidin, an antimicrobial peptide, is a family member of antibacterial and lipopolysaccharide-binding proteins mostly found in neutrophil and macrophage granules (51). This protein has been shown to complex with LDL/VLDL particles (52, 53), suggesting that VLDL may serve as a plasma reservoir of this antimicrobial protein. Proteoglycan 4 correlated with both LDL-C and triglycerides in study children. Consistent with our results, Geyer et al. (17) also reported that proteoglycan 4 was the second most strongly correlated protein with both LDL-C and triglycerides, following apoB and apoC-III, respectively, and downregulated by weight loss in obese individuals. As proteoglycan 4 is a major adipocyte extracellular matrix protein (54), it may occur in plasma from tissue leakage.

The plasma HDL proteome comprised the highest number of proteins reflecting diverse biological functions of HDL particles (8). Among 36 positive correlates of HDL-C concentrations, nine were apolipoproteins, including apoA-I. Accounting for 70% of HDL protein, apoA-I has been shown to confer anti-inflammatory, anti-oxidative, and cardioprotective properties to the lipoprotein complex (2, 4). Less abundant apolipoproteins, such as apoA-II/A-IV/C-I/C-III/D/F/M and SAA4, are also known to coregulate metabolism, including interactions with other lipoproteins and cell-specific receptors (1). PLTP is the primary protein that transfers phospholipids from triglyceride-rich lipoproteins to HDL, representing the maturation of HDL particles (55). LCAT, the extracellular cholesterol-esterifying enzyme, promotes efflux of cholesterol depots in peripheral tissues to the liver to be catabolized and excreted (56). The PON1 enzyme prevents LDL oxidation and downstream events that can form atheroma (57). Correlations with RBP4, transthyretin, and afamin, which are all known to be physically bound to HDL particles, are consistent with their known roles in HDL of transporting fat-soluble vitamins A and E (29, 58).

Interestingly, our analysis has revealed several novel correlations between HDL-C and its constituent apoA-1 with intracellular proteins (Fig. 1A). IFRD2 shared the second strongest correlation with HDL-C, although its general functions remain unknown. EIF2D, KLF17, and TMF1 are known to coregulate transcriptional and translational processes. Although these proteins are not likely carried by lipoproteins and mechanisms giving rise to these strong correlations with HDL-C in plasma are unknown, we postulate that they may regulate expression of *apoA-1* or other genes involved in HDL biogenesis and metabolism in hepatocytes (59). Lastly, positive correlations with ANXTR1 and ANXTR2, proteins found to be physically bound to HDL particles (60) and essential for homeostasis of extracellular matrix (61), could reflect HDL’s interactions with extracellular molecules and growth factors that promote resolution of inflammation and wound healing with epithelial proliferation (62).

The negative correlates of the HDL proteome are widely involved in acute phase reactions and the complement system, with more than half previously reported to be associated with inflammation (Fig. 1B, colored in red) (31). These results are consistent with evidence that HDL hosts a large number of acute phase reactants and complement factors, providing a platform for assembly of immunomodulatory complexes (7). Although these negative correlations were relatively less strong than positive correlations with HDL-C, they affirm a recent notion that HDL is an integral component of the innate host defense system (6, 8).

In this thin pediatric population, elevated plasma triglycerides and low concentrations of HDL-C were common. Similar abnormal lipid profiles have also been observed in undernourished children in Brazil, Iran, and India (63–66). Predominantly cereal-based diets with low intakes of animal source foods in this population (67) may promote hypertriglyceridemia and low HDL-C and LDL-C in these children. Subclinical inflammation can also contribute to dyslipidemia, as high triglycerides and low HDL-C concentrations were prominent in these Nepalese children with inflammation, as consistently observed in patients with diverse inflammatory disorders and infectious diseases (22) and children with cystic fibrosis (68, 69) and bacterial infections (70). Substantial inflammation-associated changes in the relative abundance of HDL-correlated proteins appear to reflect both altered lipoprotein metabolism and dysfunction. For example, increased abundance of SAA4 (+12% among inflamed children) is a hallmark of a pro-inflammatory HDL phenotype, involving a displacement of other apolipoproteins and accelerated clearance of HDL (22, 71). Reduced abundance of PLTP (−5%) and LCAT (−3%) and their potent activators (apoA-1, apoA-IV, and apoC-I) can disturb the formation and maturation of HDL (72). Reduced PON1 (−8%) can promote oxidation of LDL and lead to increased accumulation of cholesterol in macrophages (73). It has been suggested that increased abundance of acute phase proteins residing in HDL particles are part of the innate response that provides rapid protection to a host (74). However, an unresolved chronic presence of pro-inflammatory agents with loss of anti-inflammatory and anti-oxidative proteins may compromise the atheroprotective capacity of HDL (74). Changes in the abundance of proteins correlated with LDL-C (n = 1) and triglycerides (n = 4) were also observed, but to a lesser extent than the HDL proteome. As triglyceride-rich lipoproteins carry less protein cargo than HDL, elevated concentrations of plasma triglyceride in these children are more likely to be affected by other factors, such as increased de novo hepatic synthesis and secretion of VLDL and low lipoprotein lipase activity during inflammation (22).

This is the first study, to our knowledge, that has profiled plasma proteomes of lipoproteins and triglyceride in a population of undernourished children, often considered to be at low risk of dyslipidemia. Extensive data on nutritional status and subclinical inflammation enabled us to estimate the potential effects of inflammation on a family of plasma proteomes correlated with lipid and lipoproteins. An unbiased quantitative plasma proteomics approach allowed us to discover a broadened scope of proteomes by discovering novel proteins originating probably in the liver, peripheral tissues, vascular endothelium, and immune cells that may be interpreted to reflect population homeostasis of lipoproteins and their likely interactions with multiple tissues. A major limitation is that we were unable to differentiate detected proteins by whether they were physically bound to lipoprotein particles because we did not isolate and purify lipoprotein particles by ultracentrifugation (75). Another limitation of this study is that high minimum detectable limits of lipid measurement resulted in many missing values, especially in LDL-C concentrations. Lastly, due to the relative scale of protein abundance quantified by iTRAQ MS, absolute abundance of individual proteins could not be compared. As some identified proteins serve as indicators of both lipid status and likely cardioprotective or atherosclerotic function, these proteins may be good candidates for targeted quantification and further evaluation.

In this generally undernourished population of school-aged children living in the rural southern plains of Nepal, global plasma proteomic characterization revealed a family of lipoproteomes with distinct proteins correlated with concentrations of LDL-C and triglyceride, and a large highly diverse HDL proteome that displayed substantial variation by inflammation status. As dyslipidemia and chronic inflammation, both major risk factors of cardiovascular disease, may also coexist among children in similar impoverished settings, further studies are warranted to evaluate clinical and public health implications of identified proteins as early childhood biomarkers of vascular health and disease in later life.

## Supplementary Material

Supplemental Data
