# Supplementary material for: Plasma proteome correlates of lipid and lipoprotein: biomarkers of metabolic diversity and inflammation in children of rural Nepal
Source: J Lipid Res. 2018 Nov 25;60(1):149–60. doi: 10.1194/jlr.P088542 (PMC6314253; doi:10.1194/jlr.P088542)
Supplement: Supplemental Data [file supp_60_1_149__index.html]

Plasma proteome correlates of lipid and lipoprotein: Biomarkers of metabolic diversity and inflammation in children of rural Nepal — Plasma proteome correlates of lipid and lipoprotein: biomarkers of metabolic diversity and inflammation in children of rural Nepal — Supplemental Data 

# Plasma proteome correlates of lipid and lipoprotein: biomarkers of metabolic diversity and inflammation in children of rural Nepal

## Supplemental Data

- Supplemental Table S1 (.xlsx, 2.1 MB) - Supplemental Table S1
- Supplemental Table S2 (.xlsx, 297 KB) - Supplemental Table S2
